# Hf-based high-*k *materials for Si nanocrystal floating gate memories

**DOI:** 10.1186/1556-276X-6-172

**Published:** 2011-02-24

**Authors:** Larysa Khomenkova, Bhabani S Sahu, Abdelilah Slaoui, Fabrice Gourbilleau

**Affiliations:** 1 CIMAP, UMR CNRS/CEA/ENSICAEN/UCBN 6252, Ensicaen, 6 Bd Mal Juin, 14050 Caen Cedex 4, France; 2 InESS/UDS-CNRS, 23 rue du Loess, 67037 Strasbourg, France

## Abstract

Pure and Si-rich HfO_2 _layers fabricated by radio frequency sputtering were utilized as alternative tunnel oxide layers for high-*k*/Si-nanocrystals-SiO_2_/SiO_2 _memory structures. The effect of Si incorporation on the properties of Hf-based tunnel layer was investigated. The Si-rich SiO_2 _active layers were used as charge storage layers, and their properties were studied versus deposition conditions and annealing treatment. The capacitance-voltage measurements were performed to study the charge trapping characteristics of these structures. It was shown that with specific deposition conditions and annealing treatment, a large memory window of about 6.8 V is achievable at a sweeping voltage of ± 6 V, indicating the utility of these stack structures for low-operating-voltage nonvolatile memory devices.

## Introduction

In recent years, nanocrystal-based memory devices have attracted considerable attention as a possible solution to overcome the scaling issue of electronic nonvolatile memories (NVMs) http://public.itrs.net/. By using discrete nanocrystals instead of the conventional continuous floating gate as charge storage nodes, local-defect-related leakage can be reduced efficiently to improve data retention [1]. In this regard, discrete-trap type semiconductor storage materials such as Si and Ge nanocrystals (Si- and Ge-ncs) embedded in a dielectric matrix have been demonstrated as potential candidates for the fabrication of high-speed, high-density, low-power-consuming, and nonvolatile memories [2-6]. Several approaches have been reported for nanocrystal formation in a dielectric matrix, such as chemical vapor deposition, molecular beam epitaxy, or sputtering. The main attention was devoted to two major ones, namely deposition in vacuum and ion beam synthesis, since they are also used in the semiconductor industry for other purposes other than nanocrystal fabrication. Another approach for the fabrication of Si-ncs is the radio frequency (RF) magnetron sputtering, as discussed previously [7-11]. The excess Si content in the layers can be obtained by several ways. One of them is the sputtering of two separated (pure Si and SiO_2_) targets [7,8,12] or one composed (SiO_2 _target topped by Si chips) target in pure argon plasma [7,13,14]. The other one is the reactive approach, which deals with the sputtering of pure SiO_2 _target in mixed argon-hydrogen plasma [9-11] or pure Si target in argon-oxygen mixture [15]. The Si excess is controlled by varying the hydrogen [9-11] or oxygen flow rate [15] in the plasma. After subsequent high-temperature annealing, Si-ncs can be easily formed in these Si-rich SiO_2 _(SRSO) composite layers [7-15].

One of the major problems associated with the downscaling of device dimensions is the quantum tunneling limit of SiO_2_, conventionally used as a gate dielectric material in metal-oxide-semiconductor field-effect transistors. In recent studies, high-*k *gate dielectrics replaced the conventional SiO_2 _dielectric to be used as tunnel and control oxides in NVM devices, which allows for a thinner equivalent oxide thickness without sacrificing the nonvolatility [16-20]. Furthermore, the thicker physical thickness of the high-*k *dielectrics ensures good retention characteristics, while due to unique band asymmetry with Si, their lower electron barrier height allows for a larger tunneling current at low control gate voltage when the device operates in the programming regime [18,20]. In this regard, Hf-based dielectrics can be of immense interest.

In this work, different high-k/SRSO/SiO_2 _memory structures were fabricated by RF magnetron sputtering. The high-*k *layers (pure and Si-rich HfO_2_) were used as alternative tunnel layers. At the beginning, the effect of the deposition conditions and postdeposition annealing treatment was investigated separately for high-*k *and SRSO layers to obtain the optimal fabrication conditions for each material. Subsequently, the different stack structures were fabricated, and their structural and electrical properties were analyzed versus annealing treatment.

## Experimental procedure

The structures investigated in the present study were grown on p-Si (100) substrates (resistivity of approximately 15 Ω cm) by RF magnetron sputtering. Prior to deposition, the substrates were subjected to standard RCA cleaning, dipped in a diluted hydrofluoric solution (10%), dried in N_2_, and immediately transferred to the vacuum chamber of the deposition setup. Single HfO-based and SRSO layers were developed to find the optimal conditions for fabrication of p-Si/tunnel layer/charge storage layer/control layer stack memory structures.

Four-inch HfO_2 _(99.9%) and SiO_2 _(99.995%) targets were used as starting targets to grow high-*k *(HfO-based) and low-*k *(pure or Si-rich SiO_2_) layers, respectively. The HfO-based layer was grown by sputtering either pure HfO_2 _or composed HfO_2 _+ Si targets. The different Si content in the high-*k *layers was achieved by the variation of the number of Si chips topped on HfO_2 _target. In this study, Si surface ratio was *R*_Si _= 6% or 12%. The RF power density applied on HfO_2 _cathode, the argon flow, and the total plasma pressure were 0.74 W/cm^2^, 1.5 standard cubic centimeters per minute (sccm), and 40 μbar, respectively. The substrate temperatures were 45°C, 100°C, and 400°C.

The pure or Si-rich SiO_2 _layers were grown in the same chamber by sputtering of SiO_2 _target using either standard or reactive approaches. The deposition of pure SiO_2 _layers was performed in pure argon plasma (standard approach) with argon flow of 3.2 sccm. The Si-rich SiO_2 _layers were fabricated by reactive approach. The SiO_2 _target was sputtered in the mixed argon-hydrogen plasma. The argon and hydrogen flows were kept at 1.6 and 5.0 sccm, respectively. The RF power density applied on SiO_2 _cathode and the total plasma pressure were 0.74 W/cm^2 ^and 20 μbar, correspondingly, for both pure and Si-rich SiO_2 _layers. The substrate temperatures were 45°C, 100°C, and 400°C.

The deposition conditions, mentioned above, were also used for the fabrication of trilayer structures where (1) the tunnel layer is HfO-based material (either pure or Si-rich HfO_2_), (2) the charge storage layer is Si-rich SiO_2_, and (3) the control layer is SiO_2 _or HfO-based layer.

To study the effect of the postdeposition processing on the thermal stability of the high-*k *layers as well as on the formation of Si-ncs inside SiO_2 _ones, both single-layer and trilayer structures were annealed in a conventional furnace within the temperature range of 800°C to 1,100°C for 10 to 30 min under nitrogen flow. In some cases, an additional annealing in forming gas at 400°C for 60 min was also used to passivate dangling bonds, if any. After this, the Al contacts were deposited by means of thermal evaporation of Al target on the back and face sides of the structures, followed by an annealing of the final structures at 400°C for 20 min in forming gas flow.

The combination of different methods allows information about film properties to be obtained. Thus, infrared attenuated total reflectance (ATR) was used to study the structure and chemical composition of the films. ATR-FTIR spectra were measured in the range 600 to 4,000 cm^-1 ^by means of a 60° Ge Smart Ark accessory inserted in a Thermo Nicolet spectrometer (Nexus model 670) (Thermo Nicolet Corporation, Madison, USA). X-ray diffraction **(**XRD) data were obtained using a Phillips X'PERT PRO device http://www.panalytical.com/ with Cu K_α _radiation (*λ *= 0.1514 nm) at a fixed grazing angle incidence of 0.5°. An asymmetric grazing geometry was chosen to increase the volume of material interacting with X-ray beam as well as to eliminate the contribution from Si substrate. The electrical properties of the samples were studied at different frequencies using an HP 4192A LF Impedance Analyzer http://www.home.agilent.com/.

## Results and discussion

The fabrication of an NVM cell requires a perfect control of four main parameters: (1) the tunnel oxide thickness, (2) the nanocrystal density, (3) the nanocrystal diameter, and (4) the control oxide thickness. In these regards, properties of the samples were analyzed at different fabrication steps and applied to get an insight on the formation and quality of the structures. Prior to describing the electrical properties of the trilayer stack structures, let us consider separately the parameters of single pure and Si-rich HfO_2 _layers as well as Si-rich SiO_2 _layers.

### HfO-based tunnel layers

In our previous study [21], the thermal stability of amorphous structure and the chemical composition of *pure HfO*_*2 *_*layers *grown by RF magnetron sputtering after annealing at 800°C to 850°C for 15 min in nitrogen flow have been discussed in detail. Besides, the formation of monoclinic HfO_2 _phase after treatment at higher annealing temperature (*T*_A _= 900°C to 1,100°C) was also demonstrated. However, HfSiO layers were found to be stable at 950°C, whereas the increase of annealing temperature (*T*_A_) led to the formation of tetragonal HfO_2 _phase. The tetragonal phase is more preferred, since it has a higher dielectric constant (about 25). It was clearly demonstrated that the Si incorporation plays the major role for the improvement of the thermal stability of the HfO-based layers [21].

The high-frequency capacitance-voltage (C-V) study of metal-insulator-semiconductor (MIS) capacitors containing pure HfO_2 _layers (as-deposited as well as annealed at 800°C for 15 min) was performed for the samples grown at different temperatures. In most cases, the C-V curves of the annealed samples demonstrated less stretch-out effect compared to the as-deposited films due to lower number of interface states. However, the significant negative shift of flat-band voltage (*V*_fb_) up to -2 V indicates the existence of considerable amount of positive oxide charges in the films. The introduction of positive charges can be caused by the formation of SiO_*x *_interfacial layer between HfO_2 _layer and Si substrate as a result of oxygen diffusion towards Si wafer under annealing treatment [21]. In addition, the presence of oxygen vacancies inside HfO_2 _films that is most common in high-*k *gate dielectrics also gives rise to positive charge [22].

One of the limiting factors for oxygen diffusion inside HfO_2 _films can be *Si incorporation in HfO*_*2*_*-based layers*. It was supposed that due to covalent nature of Si-O bonds, the formation of oxygen interstitials and vacancies will be prevented, which in turn can give rise to an improvement in the electrical properties of high-*k *materials. In this regard, the effect of the Si content on the electrical properties of our high-*k *films was investigated.

**Figure 1:**
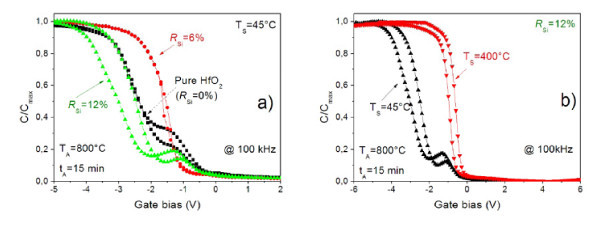
**C-V characteristics of MIS structures containing pure HfO**_**2 **_**and HfSiO films**. High-frequency C-V characteristics of pure and Si-rich HfO_2 _single layers versus Si content in the films **(a) **and deposition temperature **(b) **measured at 100 kHz. The C-V curves were normalized to their respective accumulation capacitance. All the high-*k *films were annealed at 800°C for 15 min. Deposition temperature is mentioned in the figures.

Figure 1a, b represents the C-V curves of MIS structures containing pure HfO_2 _and HfSiO films (*R*_Si _= 6% and 12%) measured at 100 kHz. As evident from the figure, pure HfO_2 _and HfSiO (*R*_Si _= 12%) layers grown at 45°C exhibit irregular C-V curves at 100 kHz. They show existence of humps, which are the characteristic features of slow traps present at the insulator/semiconductor interface, i.e., defects that are distributed away from the interface to the insulator. Hence, electron emission and capture produce broad time constant dispersion giving rise to hysteresis in the C-V curves. In addition, the C-V curves demonstrate negative *V*_fb _shift indicating the existence of fixed insulating charges in these layers. Similar effect was observed for the HfO_2_-based layers grown at 100°C (not shown here).

In contrast, HfSiO samples grown with *R*_Si _= 6% exhibit regular C-V curves. The extremely low hysteresis, along with a sharp transition from accumulation to depletion, demonstrates the high quality of interfacial as well as bulk properties of this layer.

We have further investigated the effect of deposition temperature on the C-V characteristics of HfSiO layers grown with *R*_Si _= 12%. As can be seen from Figure 1b, the samples deposited at higher temperature (*T*_S _= 400°C) show better C-V characteristics than their counterparts grown at *T*_S _= 45°C and 100°C. Therefore, we can conclude that higher deposition temperature is preferable for the Si-rich HfO_2_-based layers. Moreover, as one can see from Figure 1a, b, the C-V characteristics of HfSiO layer grown with *R*_Si _= 12% at *T*_s _= 400°C are similar to the case of HfSiO films grown at *R*_Si _= 6% and *T*_s _= 45°C. However, in the former case, the hysteresis effect is negligible compared to the last one. In this regard, one can deduce that the HfSiO layer grown with the *R*_Si _= 12% at *T*_s _= 400°C is more suitable for the fabrication of the structures even at high-temperature deposition, whereas the material with the lower Si content or pure ones can be used for low-temperature deposition approach.

Considering the above results, different types of the structures, such as HfO_2_/SRSO/HfO_2_(SiO_2_) and HfSiO/SRSO/HfSiO, were fabricated and their electrical properties were studied versus annealing treatment. Since SRSO single layers with embedded Si-ncs are considered as charge storage layers, they will be analyzed prior to describe the properties of the trilayer structures.

### SRSO single layers

The most common method to form Si-ncs entails the deposition of a thick SRSO monolayer, in which the formation of Si-ncs occurs due to phase separation on Si and SiO_2 _stimulated by high-temperature annealing. The observation of the bright photoluminescence (PL) emission in the visible spectral range is the evidence of the presence of Si-ncs. Unfortunately, the size distribution of Si-ncs in composite SRSO layers is usually broad. Thus, for the fine control of Si-ncs size the multilayer (ML) approach, where SRSO layers are alternated by SiO_2 _ones, can be applied. In this case, the control of Si-ncs occurs by means of precise thickness for SRSO layer [11,15]. In the present study, the [SRSO/SiO_2_] MLs were grown with the aim of obtaining optimal conditions for Si-ncs formation, which can be applied in future memory structures. Each ML contained 20 [SRSO/SiO_2_] periods. For all the stacks, the thickness of SiO_2 _layer was 3 nm, whereas the thickness of SRSO layer varied from 2 to 6 nm for different MLs.

**Figure 2:**
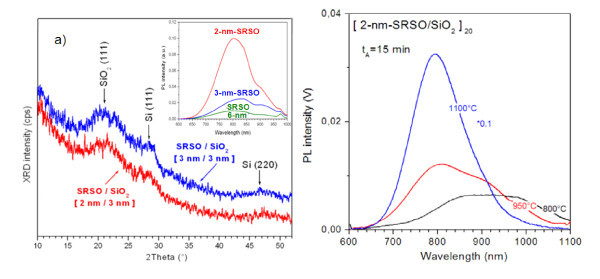
**XRD patterns and PL spectra of SRSO/SiO2 multilayers**. **(a) **GI-XRD patterns measured for [2-nm-SRSO/SiO_2_]_20 _and [3-nm-SRSO/SiO_2_]_20 _multistacks annealed at 1,100°C for 60 min. Inset, PL spectra of the same MLs. The thickness of SRSO layer for each ML is mentioned in the figure. **(b) **PL properties of the [2-nm-SRSO/SiO_2_]_20 _ML versus annealing temperature; annealing time is 15 min.

It is known that the high-temperature annealing at about 1,100°C is used to form Si-ncs required for optoelectronic application [9,11,23]. Grown MLs were annealed at 1,100°C for 60 min in nitrogen flow and were analyzed by means of XRD and PL methods to determine the formation and evolution of Si-ncs. XRD patterns taken in grazing geometry revealed the appearance of the Si-related (111) XRD peak at about 28° to 29° that confirmed the formation of Si-ncs inside the layers (Figure 2). As evident from the inset of Figure 2, the samples exhibit strong PL emission, which further confirms the formation of Si-ncs. The brightest emission was observed for the MLs with the 2-nm thickness of SRSO layer. The increase of the thickness of SRSO layer, leading to the increase of Si-ncs average size, results in the shift of PL peak position to the higher wavelength side (inset of Figure 2).

It is worth to note that pure HfO_2 _material does not conserve amorphous structure upon an annealing at high temperatures (900°C to 1,100°C). Such treatment results in the formation of monoclinic HfO_2 _phase in the single layers [21]. The appearance of grain boundaries can significantly degrade electrical properties of the structures. Thus, this dictates the elaboration of the lower thermal budget conditions for the formation of Si-ncs. In this regard, grown SRSO/SiO_2 _MLs were also annealed at relatively lower temperatures (800°C to 950°C) for 10 to 15 min. For all the cases, PL emission was obtained. However, the brightest light emission was found for [2-nm-SRSO/SiO_2_]_20 _ML structure (Figure 2b). Its PL spectrum is narrower than that observed for [6-nm-SRSO/SiO_2_]_20 _ML. It is obvious that the phase separation can occur easily for thinner SRSO layers due to smaller Si diffusion path, and this can explain the narrower PL band for [2-nm-SRSO/SiO_2_]_20 _ML, confirming the narrower size distribution of Si-ncs. The decomposition process usually completes faster for thinner SRSO layer, resulting in the formation of Si-ncs and SiO_2 _phase (instead of SiO_*x *_one). So, the formation of Si-ncs/SiO_2 _barrier instead of Si-ncs/SiO_*x *_is more probable for thinner layers. Additionally, such layers are more preferable for obtaining better electrical properties.

Based on the abovementioned results, the deposition and postdeposition conditions elaborated for single HfO-based layers and for SRSO/SiO_2 _MLs were adopted for the fabrication of trilayer structures, in which SRSO layer plays the role of charge storage layer. Low thermal budget was applied for the SRSO layers to form Si-ncs accompanied by the conservation of the amorphous nature of HfO-based layer.

### The electrical properties of the structures

#### HfSiO/SRSO/HfSiO

**Figure 3:**
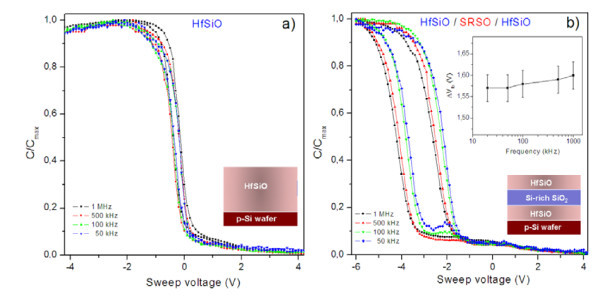
**C-V data of single HfSiO layer and HfSiO/SRSO/HfSiO structure measured at different frequencies**. Comparison of C-V data for single HfSiO layer **(a) **and HfSiO/SRSO/HfSiO structure **(b) **measured at different frequencies. *R*_Si _= 12%. Annealing treatment at *T*_A _= 950°C, *t*_A _= 15 min, N_2 _flow. Inset of figure (b) demonstrates variation of Δ*V*_fb _versus applied frequency at 6 V sweep voltage.

First of all, let us consider electrical properties of HfSiO/SRSO/HfSiO (or SiO_2_) structures. As it was mentioned above, these structures can be fabricated at higher temperatures since HfSiO layers conserve their amorphous structure at *T*_S _= 400°C to 500°C and *T*_A _= 950°C. Thus, the annealing was performed at *T*_A _= 800°C to 1,100°C for *T*_A _= 15 min in nitrogen flow to obtain the information about memory effect caused by Si-ncs. As one can see from the Figure 3a, C-V curves of Al/HfSiO/Si capacitor structures show a sharp transition from accumulation to inversion, indicating a low density of interface states in the samples under study. The MIS structures show negligible hysteresis loop. In contrast, Al/HfSiO/SRSO/HfSiO/p-Si memory structures exhibit significant counterclockwise hysteresis loop, and the memory window (Δ*V*_fb_) was estimated to be approximately 1.7 V from flat-band voltage values. The counterclockwise nature of C-V curves is generally attributed to charge storage through substrate injection mechanism. When a positive bias voltage is applied, electrons are being injected from the inversion layer of the Si substrate into the gate dielectric matrix. When a negative voltage is applied, electrons are ejected back into the Si substrate (equivalent to hole injection from the deep accumulation layer of the substrate), resulting in a shift of the C-V curve towards negative voltages. It is interesting to note that the C-V curves of Al/HfSiO/SRSO/HfSiO/p-Si memory structures shift towards more positive bias with decreasing frequency, and the shift is more prominent in the low frequency region. The shift is marked by minimal frequency dispersion in accumulation, capacitance indicating minimal influence of series resistance, and dielectric constant variation with altering the measurement frequency. From the inset of Figure 3b, it is noteworthy that the same amount of hysteresis and stored charge was obtained irrespective of the measurement frequency. Hence, the capacitance shift can be attributed to the presence of fast traps and/or border traps (near-interfacial traps), which can have a rapid communication with the underlying Si substrate [24]. From all these observations, we can ascertain that the observed memory window is predominantly due to the formation of Si-ncs. It should be noted that an annealing at 950°C for 15 min was found to provide the highest Δ*V*_fb _value, whereas the increase or decrease of *T*_A _results in the essential decrease of Δ*V*_fb_. For lower annealing temperatures, this effect can be due to noncompleted phase separation within the SRSO layer. For higher annealing temperatures, complete oxidation of SRSO layer should occur. Besides, the phase separation inside HfSiO layers can occur as it was demonstrated in [25].

#### HfO_2_/SRSO/SiO_2 _structures

The trilayer structures with the fixed thicknesses of tunnel (4 nm) and control (10 nm) layers and different thicknesses of SRSO layer (from 2 to 4 nm) were studied versus annealing treatment. As it was shown above, for all of them, the formation of Si-ncs is expected upon annealing. Going further, it is worth to note that the best electrical properties were demonstrated by the structures with 2-nm-thick SRSO layers, and they will be discussed below.

**Figure 4:**
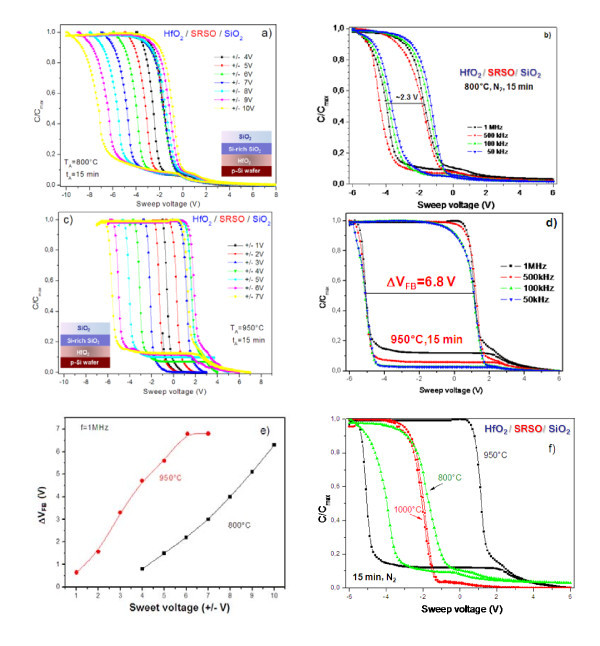
**C-V characteristics of annealed HfO**_**2**_**/SRSO/SiO**_**2**_. C-V characteristics of HfO_2_/SRSO/SiO_2 _annealed at 800°C for 15 min **(a, b) **and at 950°C for 15 min **(c, d) **measured at 1 MHz (a, c) and versus frequency measured at 6 V sweep voltage (b, d). **(e) **The variation of Δ*V*_fb _versus sweep voltage for two annealing temperatures; **(f) **the comparison of C-V curves measured at 1 MHz versus annealing temperature. Annealing time is 15 min for all the figures.

Figure 4a shows the C-V curves of HfO_2_/SRSO/SiO_2 _stack structures annealed at 800°C for 15 min in the MIS structure taken at various sweep voltages. The hysteresis memory window increases from approximately 1 V to approximately 6 V with increasing the sweep voltage from ± 4 to ± 10 V. The counterclockwise nature of the hysteresis loop indicates net electron trapping in the MIS capacitor. However, frequency-dependent C-V curves show nonparallel shifts with varying measurement frequency, indicating the presence of some interfacial traps and/or border tarps in the MIS capacitor. We speculate that the charge trapping is due to near interfacial traps and excess of silicon at SRSO/SiO_2 _and SRSO/HfO_2 _interfaces rather than Si-ncs.

The annealing temperature was further increased to 950°C (keeping the same annealing time as 15 min), and significant charge storage was achieved at relatively lower sweep voltages. The frequency dependent C-V curves were found to be almost constant within the frequency range of 1 MHz to 10 kHz. All of them show a sharp transition from accumulation to inversion region, indicating less number of interfacial traps. The memory window value increases from Δ*V*_fb _= 1 V to Δ*V*_fb _= 7 V with increasing the sweep voltage from ± 1 to ± 7 V. It is worth noting that with annealing temperature increasing from 800°C to 950°C, the Δ*V*_fb _value increases from 2.3 to 6.8 V at a sweep voltage of ± 6 V. This can be due to the increase of the Si-ncs number and to the better performance of surrounding SiO_2 _matrix. The latter favors the formation of the higher barrier for carrier tunneling from gate contact. However, annealing at *T*_A _> 950°C results in a decrease of Δ*V*_fb _value to 0.05 V (Figure 4f). This can be caused by complete oxidation of SRSO layer without formation of Si-ncs and by Si out-diffusion from the SRSO layer through the HfO_2 _layer [22,23].

Considering the results presented above, the investigation of the structures by means of ATR and XRD methods was performed to obtain the information about transformation of SRSO layer as well as about the nature of HfO_2 _tunnel layer. In the last case, this was impacted by the fact that an annealing at temperatures higher than 800°C could not favor the stability of HfO_2 _amorphous structure. It is more probable that the crystallization of HfO_2 _layer occurred. However, the ATR spectra did not reveal any formation of monoclinic HfO_2 _phase, since HfO vibration band was found to be featureless. At the same time, the XRD study showed that at *T*_A _> 900°C, the formation of tetragonal HfO_2 _phase occurs, while at lower *T*_A_, the tunnel layer conserves its amorphous structure (not shown here). However, the determination of Si-ncs by this method met some difficulties due to overlapping of the peaks from tetragonal HfO_2 _phase and Si-ncs. The TEM observation of the abovementioned samples is currently under investigation to get a clear picture about the evolution of Si-ncs and HfO_2 _layers. However, we can conclude that for *T*_A _= 950°C, the memory effect is predominantly due to Si-ncs formation.

## Conclusion

The application of pure HfO_2 _and HfSiO layers fabricated by RF magnetron sputtering as alternative tunnel layers for high-*k*/Si-ncs-SiO_2_/SiO_2 _memory structures is demonstrated. The effect of the Si incorporation of the electrical properties of high-*k *layers was investigated. It is shown that there is an optimal Si content allowed to obtain desirable C-V parameters for single layers. The Si-rich SiO_2 _layers were used as charge storage layers, and their properties were studied versus deposition conditions and annealing treatment. The C-V measurements of fabricated stack structures show that with specific deposition conditions and annealing treatment, a large memory window (about 6.8 V) is achievable at a sweeping voltage of ± 6 V, indicating the utility of these stack structures for low-operating-voltage NVM devices.

## Competing interests

The authors declare that they have no competing interests.

## Authors' contributions

LK designed the study, fabricated the samples investigated and performed post-fabrication treatment, carried out the characterization studies and analyzed the results, and prepared the draft of the manuscript. BSS carried out electrical characterization of the samples and performed the analysis of the results. AS and FG participated in the coordination of study. All authors read and approved the final manuscript.
